# Abdominal Pain after Gastric Bypass: Labor, Uterine Rupture, or Obstruction and Internal Hernia

**DOI:** 10.1155/2011/415795

**Published:** 2011-11-24

**Authors:** Sarah N. Cross, Unzila Nayeri, Andrew Duffy, Christian M. Pettker

**Affiliations:** ^1^Department of Obstetrics, Gynecology & Reproductive Sciences, Yale School of Medicine, Yale University, 333 Cedar Street, P.O. Box 208063, New Haven, CT 06520-8063, USA; ^2^Department of Surgery, Yale University School of Medicine, New Haven, CT 06510, USA

## Abstract

*Background*. Although gastric bypass may reduce obesity-related complications of subsequent pregnancies, surgical complications requiring antenatal and postpartum interventions are not uncommon. *Case*. A 26-year-old G4P1112 status post-Roux-en-Y gastric bypass required multiple urgent antenatal evaluations due to frequent episodes of abdominal pain. At 35 + 4 weeks, she presented with severe abdominal pain; initial evaluation was negative for gastrointestinal pathology. The patient was found to be in preterm labor and underwent a repeat cesarean section. The postoperative course was complicated by bowel obstruction due to internal hernia resulting in an emergent laparotomy and a prolonged hospital course. *Conclusion*. As more reproductive-aged women opt for surgical treatment of obesity, it is essential that obstetricians recognize complications to be able to counsel and appropriately care for these patients.

## 1. Introduction

As the prevalence of obesity continues to increase, complications of obesity during pregnancy—namely, gestational diabetes, hypertensive disorders of pregnancy, infections, postpartum hemorrhage, fetal anomalies, cesarean sections, and birth injuries—will become more common. A 1991 NIH consensus statement recommended that the minimum criteria for considering bariatric surgery include BMI greater than 40 kg/m^2^ and BMI of 35–40 kg/m^2^ in the setting of medical comorbidities [1]. An estimated 100,000–120,000 weight loss procedures were performed in 2005 [2]. The majority of these procedures (approximately 73%) are performed on women, with reproductive aged-women comprising the bulk of these patients [3]. Weight loss after gastric bypass decreases the rate of certain comorbidities including gestational diabetes, gestational hypertension, preeclampsia, macrosomia, and cesarean delivery [4–6]. More importantly, pregnancy after gastric bypass surgery has been found to be safe with similar rates of perinatal complications to nonobese patients [6].

Complications of roux-en-Y gastric bypass (RYGB) include bowel obstruction, closed loop obstruction, bowel infarction, ischemia, necrosis or perforation, volvulus, intussusceptions, and internal hernia, all of which can occur and require surgical intervention in or after pregnancy [6, 7]. Additional fetal and maternal death has been reported [8]. We describe a case of pregnant patient status after gastric bypass complicated by a hernia and obstruction discovered soon after cesarean delivery.

## 2. Case Presentation

A twenty-six-year-old Gravida 4 Para 1112 at 35 + 4 weeks' gestational age with a history of morbid obesity (BMI 41 kg/m^2^) status-post-laparoscopic RYGB at an outside institution thirteen months prior to conception, presented to Labor and Delivery with severe abdominal pain. With a weight loss of 80 pounds following her RYGB, her type II diabetes mellitus (DM) had resolved and her prepregnancy BMI was 26.5 kg/m^2^. Although she had minimal followup with her surgeon and antenatally had been noncompliant with diet and exercise, she was on monthly vitamin B12 injections during her pregnancy. Her past surgical history was also significant for a laparoscopic appendectomy prior to her RYGB. The patient's obstetrical history was significant for one full-term cesarean delivery due to arrest of descent, a repeat cesarean delivery at 35 + 6 weeks in the setting of painful contractions, and one first trimester termination. 

The patient's antenatal course was complicated by persistent abdominal pain for which she was evaluated urgently six times during her pregnancy, occasionally associated with nausea and emesis. At 26 weeks' gestational age, she had an MRI of the abdomen and pelvis, which was negative for acute pathology. At 35 + 4 weeks' gestation, the patient represented to the triage unit with sudden-onset epigastric pain radiating to her back. She denied nausea, vomiting, fevers, chills, and contractions. The patient was afebrile with stable vital signs. On exam, the midepigastric area was notable for moderate tenderness to palpation and guarding without rebound. The lower abdomen was nontender. The fetal heart tracing was category I, and the tocodynometer revealed uterine contractions every three minutes. The cervix was long, closed, and posterior. Laboratory values, including white blood cell count, liver function tests, pancreatic enzymes, and lactate, were all within normal limits. The urinalysis was remarkable for a specific gravity of 1.025 and small ketones. Plain films did not demonstrate presence of free air. An emergency general surgery consult was obtained, and the surgeons felt that the patient's presentation was unrelated to her prior RYGB. Upon repeat examination, the cervix was dilated to two centimeters. 

With the diagnosis of abdominal pain and uncomfortable preterm contractions, perhaps indicating a compromised uterine scar in the setting of two prior cesarean deliveries, the patient underwent a repeat cesarean section. Due to dense adhesions between the rectus muscles and the lower uterine segment, a classical uterine incision was performed via a Pfannenstiel skin incision. A 4 × 5 cm uterine window was noted at the level of the lower uterine segment. The patient delivered a 2730 gram male infant with Apgars of 9 and 9 at 1 and 5 minutes, respectively. The estimated blood loss was 1150 mL, and a Jackson-Pratt drain was placed in the pelvis. Pathologic examination of the placenta revealed no abnormalities. The operative team presumed the etiology of her pain was preterm labor and a uterine dehiscence. Complete abdominal exploration was not performed.

After delivery the patient's abdominal pain initially improved. On postoperative day (POD) 2, the patient complained of nausea and clear emesis but was passing flatus. On POD 3, the patient was unable to tolerate a clear diet. An abdominal radiograph revealed minimally dilated loops of small bowel with air-fluid levels in the colon without free air. On POD 4, the patient had feculent emesis and an elevated lactic acid to 3.3 mmol/L. A CT scan of the abdomen and pelvis showed a left-sided closed loop obstruction as well as an obstruction of the Roux loop at the level of the jejunostomy. There was no evidence of free air. The patient was taken to the operating room by a bariatric surgeon for an operative laparoscopy, which was converted to a laparotomy. Intraoperative findings revealed a chronic internal hernia through Peterson's space in addition to a closed loop obstruction of the Roux limb. The jeujeunostomy had been created on the right side leaving the biliary limb running posterior to the roux limb causing an anatomic chronic intermittent obstruction that had progressively worsened during pregnancy. A complete revision of the RYGB was performed with two small bowel resections, including areas of full-thickness necrosis and three new anastamoses. Peterson's space was reapproximated to prevent hernia recurrence leaving the patient with a long limb bypass anatomy. The patient's postoperative course was complicated by aspiration pneumonia and a prolonged intubation. The patient was extubated on POD 9 and discharged home on POD 13 from the laparotomy. She did not present for her postpartum visit but did have a normal postoperative followup with her bariatric surgeon. 

## 3. Discussion

In addition to vitamin and mineral deficiencies and anemia, surgical complications of RYGB are not uncommon during pregnancy. Several case reports have described complications such as volvulus and internal hernias either discovered during cesarean section or occurring postcesarean delivery. We present a case with chronic symptoms of internal hernia for much of her pregnancy. At the time of delivery, these symptoms may have been confused for preterm contractions and abdominal pain attributed to possible uterine rupture.

It has previously been suggested that surgical exploration not be delayed in the absence of positive findings on imaging [7]. Delays in initiating or completing a workup contribute further to adverse outcome in these cases. Although our patient had an MRI at 26 weeks' gestation that did not show abnormalities, she was likely in the process of developing an internal hernia and/or obstruction. Indeed, this may have been occurring at the time of presentation for delivery or even been the precipitating event for her preterm labor. Recognizing the quality of her abdominal pain and episodic urgent-care visits in the pregnancy, evaluation around the time of delivery for a complication related to the RYGB may have avoided a delay in diagnosis. Our patient had several areas of full-thickness bowel necrosis, and, therefore, would have benefited from surgical exploration either in the midtrimester or at the time of delivery instead of the delay of five days.

While other case reports have described resorting to delivery to facilitate surgical exploration, we describe a case of preterm labor complicating a gastric bypass patient's presentation of abdominal pain. Our case demonstrates that pregnancy-specific conditions, like preterm labor and cesarean scar dehiscence, can obscure the evaluation and diagnosis of complications associated with RYGB. It is, therefore, essential to entertain a broad differential diagnosis when caring for pregnant patients status post-gastric-bypass. Obstetricians should consider abdominal exploration, intraoperative surgery consult, or a planned procedure with general surgery following delivery when patients present with abdominal pain not entirely consistent with an obstetric diagnosis. As obesity continues to be a dominant comorbidity and reproductive-aged women opt for surgical treatment prior to pregnancy, it is paramount that obstetricians recognize and understand the possible antepartum and postpartum complications so that they are able to counsel and appropriately care for these patients.
